# Nanobody-based pannexin1 channel inhibitors reduce inflammation in acute liver injury

**DOI:** 10.1186/s12951-023-02137-1

**Published:** 2023-10-11

**Authors:** Raf Van Campenhout, Timo W. M. De Groof, Prashant Kadam, Brenda R. Kwak, Serge Muyldermans, Nick Devoogdt, Mathieu Vinken

**Affiliations:** 1 Entity of In Vitro Toxicology and Dermato-Cosmetology, Department of Pharmaceutical and Pharmacological Sciences, Vrije Universiteit Brussel, 1090 Brussels, Belgium; 2 In Vivo Cellular and Molecular Imaging Laboratory, Department of Molecular Imaging, Vrije Universiteit Brussel, Laarbeeklaan 103, 1090 Brussels, Belgium; 3 Department of Pathology and Immunology, Faculty of Medicine, University of Geneva, CH-1211 Geneva, Switzerland; 4 Geneva Center for Inflammation Research, Faculty of Medicine, University of Geneva, CH-1211 Geneva, Switzerland; 5 Laboratory of Cellular and Molecular Immunology, Bioengineering Sciences Department, Vrije Universiteit Brussel, 1050 Brussels, Belgium

**Keywords:** Acute liver disease, Inflammation, Nanobody, Pannexin1, Therapy

## Abstract

**Background:**

The opening of pannexin1 channels is considered as a key event in inflammation. Pannexin1 channel-mediated release of adenosine triphosphate triggers inflammasome signaling and activation of immune cells. By doing so, pannexin1 channels play an important role in several inflammatory diseases. Although pannexin1 channel inhibition could represent a novel clinical strategy for treatment of inflammatory disorders, therapeutic pannexin1 channel targeting is impeded by the lack of specific, potent and/or in vivo-applicable inhibitors. The goal of this study is to generate nanobody-based inhibitors of pannexin1 channels.

**Results:**

Pannexin1-targeting nanobodies were developed as potential new pannexin1 channel inhibitors. We identified 3 cross-reactive nanobodies that showed affinity for both murine and human pannexin1 proteins. Flow cytometry experiments revealed binding capacities in the nanomolar range. Moreover, the pannexin1-targeting nanobodies were found to block pannexin1 channel-mediated release of adenosine triphosphate. The pannexin1-targeting nanobodies were also demonstrated to display anti-inflammatory effects in vitro through reduction of interleukin 1 beta amounts. This anti-inflammatory outcome was reproduced in vivo using a human-relevant mouse model of acute liver disease relying on acetaminophen overdosing. More specifically, the pannexin1-targeting nanobodies lowered serum levels of inflammatory cytokines and diminished liver damage. These effects were linked with alteration of the expression of several NLRP3 inflammasome components.

**Conclusions:**

This study introduced for the first time specific, potent and in vivo-applicable nanobody-based inhibitors of pannexin1 channels. As demonstrated for the case of liver disease, the pannexin1-targeting nanobodies hold great promise as anti-inflammatory agents, yet this should be further tested for extrahepatic inflammatory disorders. Moreover, the pannexin1-targeting nanobodies represent novel tools for fundamental research regarding the role of pannexin1 channels in pathological and physiological processes.

**Graphical Abstract:**

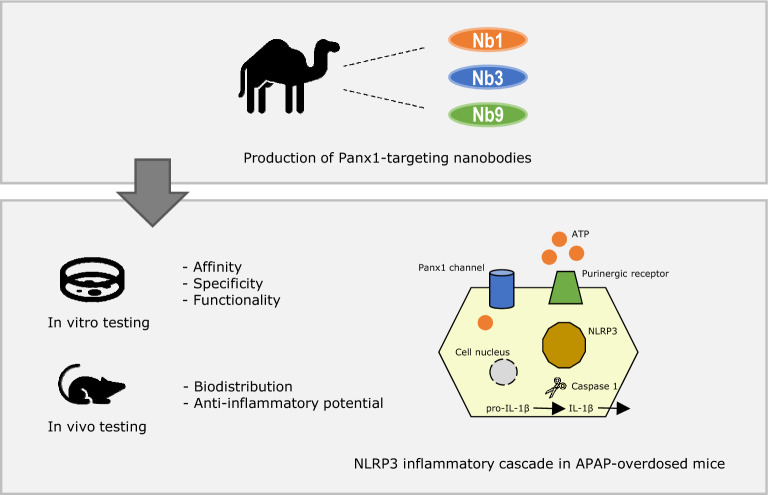

**Supplementary Information:**

The online version contains supplementary material available at 10.1186/s12951-023-02137-1.

## Background

Drug-induced liver injury  (DILI) is a major cause of all clinical cases of acute liver disease [1]. An overdose of acetaminophen (APAP) is the main reason of DILI in many countries worldwide [2–4]. This analgesic and antipyretic drug causes severe or fatal liver injury through accumulation of *N*-acetyl-*p*-benzoquinone imine (NAPQI), a metabolite of APAP. Disproportionate amounts of NAPQI trigger mitochondrial dysfunction and DNA damage, which causes necrotic hepatic cell death. The latter evokes an inflammatory response in liver [5]. In this respect, APAP-induced hepatotoxicity increases serum levels of multiple cyto- and chemokines, such as interleukin (IL)-6, IL-8, IL-10 and monocyte chemoattractant protein-1 (MCP-1) in human patients [6–8].

Pannexin1 (Panx1) proteins are identified as important actors in liver injury triggered by APAP [9, 10]. Panx1 proteins form water-filled channels at the cell plasma membrane surface. They drive various physiological processes via trafficking of ions, such as potassium, and small molecules, including adenosine triphosphate (ATP) [11]. During inflammation, various stimuli, including changes in extracellular potassium concentration and phosphorylation, induce the opening of Panx1 channels leading to the extracellular release of ATP and metabolites [12, 13]. A variety of Panx1-targeting agents, such as small molecules, peptides and monoclonal antibodies, has been proposed for the pharmacological closing of Panx1 channels [14–17]. However, these compounds suffer from lack of affinity, specificity, potency and/or stability. In this study, we describe the generation of nanobodies, antibody fragments derived from heavy chain-only antibodies, targeting Panx1 proteins. These newly developed Panx1-targeting nanobodies bind and inhibit Panx1 channel activity resulting in anti-inflammatory effects. Moreover, nanobody treatment reduced inflammation in a mouse model of acute liver disease. This suggests a role for these novel Panx1-targeting nanobodies for the treatment of acute liver disease and potentially of other inflammatory diseases.

## Methods

### DNA constructs

Plasmids: packaging plasmid pCMVΔR8.9 (Addgene plasmid 12263, Trono Lab), VSV.G encoding plasmid pMD.G (Addgene plasmid 12259, Trono Lab), pRP and pASIET vectors (Addgene plasmid 17448, Trono Lab) harbouring mouse Panx1 (mPanx1) or human Panx1 (hPanx1) coding sequence, pMECS and pHEN6c vectors.

### Cell cultures

SV40 immortalised DUBCA fibroblasts (*Camelus dromedarius*) and RAW264.7 murine macrophages were cultured in Dulbecco’s modified Eagle’s medium (Thermo Fisher Scientific, Belgium) supplemented with 10% v/v fetal bovine serum (FBS) (Thermo Fisher Scientific), 50 µg/mL streptomycin sulphate (Merck, Germany) and 7.33 I.E./mL sodium benzyl penicillin (Continental Pharma, Belgium), at 37 °C with a constant supply of 5% CO_2_.

Human embryonal kidney (HEK) 293 T cells were cultured in Dulbecco’s modified Eagle’s medium supplemented with 0.110 g/L sodium pyruvate (Merck), 0.328 g/L L-glutamine (Merck), 10% v/v FBS, 50 µg/mL streptomycin sulphate and 7.33 I.E./mL sodium benzyl penicillin, at 37 °C with a constant supply of 5% CO_2_.

*Escherichia coli* TG1 cells were cultured in 2xTY medium (*i.e.* 16 g tryptone (Duchefa Biochemie, Netherlands), 10 g yeast extract (Duchefa Biochemie) and 5 g NaCl (Thermo Fisher Scientific) dissolved in 1 L water). For the preparation of electrocompetent *Escherichia coli* TG1 cells, an overnight TG1 cell culture was grown until OD_600nm_ values of 0.8–1.0 were reached. Cells were placed on ice for 1 h and centrifuged at 2200 × *g* for 7 min at 4 °C. Pelleted cells were resuspended in ice-cold water and once again centrifuged at 2200 × *g* for 7 min at 4 °C, supernatant was removed and cells were resuspended in ice-cold glycerol solution, water supplemented with 10% v/v glycerol (Duchefa Biochemie).

*Escherichia coli* WK6 cells were cultured in TB medium (*i.e.* 2.3 g KH_2_PO_4_ (Merck), 16.4 g K_2_HPO_4_.3H_2_O (Merck), 12 g tryptone, 24 g yeast extract and 0.4% v/v glycerol dissolved in 1 L water). For the preparation of electrocompetent *Escherichia coli* WK6 cells, the same procedure was used as for TG1 cells.

### Animal studies

Male C57BL/6 J mice (Charles River Laboratories, France) of approximately 2 months of age were used and housed in the animal facility of the Faculty of Medicine and Pharmacy of the Vrije Universiteit Brussel-Belgium. All animals were kept under controlled environmental conditions (19–23 °C, 30–70% relative humidity, 14/10 h light/dark cycle) with free access to food and water. Protocols to examine the in vivo biodistribution of Panx1-targeting nanobodies and anti-inflammatory effects of Panx1-targeting nanobodies have been approved by the local Ethical Committee of the Vrije Universiteit Brussel-Belgium (project numbers 21-210-1 and 20-210-8). All animals received daily follow-up by animal care takers or veterinarians according to the criteria by the guidelines provided by the Vrije Universiteit Brussel-Belgium.

### Generation of Panx1-overexpressing DUBCA cells

To obtain cells overexpressing mPanx1 or hPanx1, DUBCA cells were lentivirally transduced. A transient transfection mixture was prepared by adding 15 µg pMD.G, 30 µg pCMVΔR8.9 and 45 µg transfer plasmid (pASIET vectors harbouring mPanx1 or hPanx1 coding sequence) to 5 mL of Opti-Minimal Essential Medium (Thermo Fisher Scientific). A polyethylenimine (PEI) mixture of 180 µg PEI (Polysciences, Germany) in 5 mL Opti-Minimal Essential Medium was made. The PEI mixture was added to the transfection mixture, vortexed and incubated at room temperature for 30 min. This transfection-PEI mixture was added to HEK 293 T cells and incubated for 4 h at 37 °C with CO_2_ supply. After 4 h, the transient transfection mixture was removed and replaced with cell culture medium for 48 h. Medium was harvested and lentiviral particles were concentrated in 10 µg/mL protamine sulfate (LEO Pharma, Belgium) enriched-PBS solution upon ultracentrifugation at 20,000 × *g* for 5 min. Next, DUBCA cells were transduced with lentiviral particles at a multiplicity of infection of 20 for 72 h. Transduced cells are referred to as DUBCA mPanx1 and DUBCA hPanx1, untransduced cells are named as DUBCA wild-type (WT) cells.

### In vitro characterisation of DUBCA Panx1 cells

For immunoblot analysis, DUBCA WT, DUBCA mPanx1 and DUBCA hPanx1 cells were harvested from culture flasks (Corning, USA) by dissociation with TrypLE (Thermo Fisher Scientific). Proteins were isolated by homogenising cell pellets in radioimmunoprecipitation (RIPA) buffer (Thermo Fisher Scientific) supplemented with 1% v/v ethylenediaminetetraacetic acid (EDTA) solution (Thermo Fisher Scientific) and 1% v/v protease and phosphatase inhibitor cocktail (Thermo Fisher Scientific). Samples were mixed and placed on ice for 20 min. Thereafter, cell lysates were centrifugated at 14,000 × *g* for 20 min and proteins in supernatants were collected. Protein concentration of each sample was determined using a bicinchoninic acid (BCA) Protein Assay Kit (Thermo Fisher Scientific). Proteins were fractionated on sodium dodecyl sulphate (SDS) polyacrylamide gels (Bio-Rad Laboratories, USA) and blotted afterwards onto a nitrocellulose membrane (Bio-Rad Laboratories). Membranes were blocked with blocking buffer, 5% w/v non-fatty milk powder (Régilait, France) in Tris-buffered saline solution (*i.e.* 20 mM Tris (Merck) and 135 mM NaCl (Merck)) containing 0.1% v/v Tween-20 (Merck) (TBS/T), for 1 h at room temperature. After blocking, membranes were incubated with a primary antibody directed against Panx1 (Clone D9M1C, Cell Signaling Technology, USA) (Table 1) and washed 3 times with TBS/T. Thereafter, membranes were incubated with a horseradish peroxidase-conjugated secondary antibody (Clone P0448, Dako, USA) (Table 1). Membranes were washed 3 times with TBS/T and detection of Panx1 proteins was carried out by means of an enhanced chemiluminescence Western Blotting Substrate Kit (Thermo Fisher Scientific) and a ChemiDoc MP Imaging System (Bio-Rad Laboratories). Panx1 signals in DUBCA mPanx1 and DUBCA hPanx1 cells were normalised against total protein loading and expressed as relative alterations compared to DUBCA WT cells using Image Lab software (Bio-Rad Laboratories).

**Table 1:** Overview antibodies

Antibody	Dilution	Incubation period	Incubation temperature
In vitro characterisation of DUBCA Panx1 cells (immunoblotting)			
Clone D9M1C (Panx1) (Cell Signaling Technology)	1/500 in blocking buffer	24 h	4 °C
Goat anti-rabbit horseradish peroxidase-conjugated antibody (Clone P0448) (Dako)	1/500 in blocking buffer	1 h	Room temperature
In vitro characterisation of DUBCA Panx1 cells (immunocytochemistry)			
Clone ABN242 (Panx1) (Merck)	1/250 in permeabilization buffer	24 h	4 °C
Donkey anti-rabbit Alexa Fluor^®^ 594-conjugated antibody (Clone A-21207) (Thermo Fisher Scientific)	1/250 in permeabilization buffer	1 h	Room temperature
Flow cytometry with Panx1-targeting nanobodies			
Hemagglutinin Alexa Fluor^®^ 488-conjugated antibody (Clone 901509) (BioLegend)	1/1000 in flow cytometry buffer	20 min	4 °C
In vitro inflammation assay			
Clone ab254360 (IL-1β) (Abcam)	1/500 in blocking buffer	24 h	4 °C
Goat anti-rabbit Alexa Fluor^®^ 488-conjugated antibody (Clone ab150077) (Abcam)	1/500 in blocking buffer	1.5 h	4 °C
Immunoblot analysis of liver proteins			
Clone sc-514 (caspase-1) (Santa Cruz)	1/500 in blocking buffer	24 h	4 °C
Clone HPA009128 (CYP2E1) (Atlas Antibodies)	1/500 in blocking buffer	24 h	4 °C
Clone ab254360 (IL-1β) (Abcam)	1/500 in blocking buffer	24 h	4 °C
Clone ab263899 (NLRP3) (Abcam)	1/500 in blocking buffer	24 h	4 °C
Clone D9M1C (Panx1) (Cell Signaling Technology)	1/500 in blocking buffer	24 h	4 °C
Goat anti-mouse horseradish peroxidase-conjugated antibody (Clone P0447) (Dako, USA)	1/1000 in blocking buffer	1 h	Room temperature
Goat anti-rabbit horseradish peroxidase-conjugated antibody (Clone P0448) (Dako, USA)	1/1000 in blocking buffer	1 h	Room temperature

The table lists the different types of antibodies used in this research paper

Immunocytochemistry analysis was performed by seeding DUBCA WT, DUBCA mPanx1 and DUBCA hPanx1 cells at a density of 25,000 cells/well in 750 µL/well of cell culture medium using a 24-well cell culture plate (Corning). The next day, supernatant was aspirated and cells were fixed with 4% w/v paraformaldehyde (Polysciences) in PBS for 15 min at room temperature. Next, DUBCA cells were washed 3 times with PBS and incubated with permeabilization buffer, PBS enriched with 0.1% v/v Triton X-100 (Thermo Fisher Scientific), for 10 min at room temperature. Subsequent blocking of the cells was performed with blocking buffer, permeabilization buffer containing 0.75% w/v glycine (Merck) and 2% w/v bovine serum albumin (BSA) (Merck). Cells were blocked for 15 min at room temperature followed by incubation with a primary antibody directed against Panx1 (Clone ABN242, Merck) (Table 1). Cells were washed 3 times with PBS and incubated with an Alexa Fluor^®^ 594-conjugated secondary antibody donkey anti-rabbit (Clone A-21207, Thermo Fisher Scientific) (Table 1). After 3 times washing with PBS, nuclear counterstaining was performed with Hoechst solution (H3570) (Thermo Fisher Scientific) and Vectashield (Vector laboratories, USA) was used as mounting medium. For negative controls, an identical procedure was followed, but the primary antibody was omitted. Detection was performed using a fluorescence microscope Nikon Eclipse Ti equipped with a 20 × objective (Nikon, Japan).

### Identification of Panx1-targeting nanobodies

A llama (*Lama glama*) (Lamasté, Belgium) was immunised 4 times at biweekly intervals with 2 mg of pRP plasmids expressing the mPanx1 gene and subsequently boosted 2 times with 2.10^7^ DUBCA cells overexpressing mPanx1. Following immunisation, anticoagulated blood from the jugular vein was taken to collect RNA from blood lymphocytes. This protocol has been approved by the local Ethical Committee of the Vrije Universiteit Brussel-Belgium (project number 16-601-1). RNA was extracted using a RNeasy mini Kit (Qiagen, Germany) and reverse transcribed to cDNA using the SuperScript II First-Strand Synthesis System for reverse transcription polymerase chain reaction (Thermo Fisher Scientific). Nanobody coding sequences were retrieved from this cDNA pool using the nested PCR principle. Application of sequentional amplification reactions also introduced restriction enzyme sites at the 5′ and 3′ ends of the nanobody coding sequences for cloning into the pMECS vector. A 3:1 molar ratio of insert to vector was used to ligate the nanobody coding sequence into the vector. Electrocompetent *Escherichia coli* TG1 cells were transformed with the ligated material and grown for 24 h to construct a library of nanobodies of 10^7^ individual transformants. M13K07 helper phages were added at multiplicity of infection of 20 for 30 min to display the nanobodies at the tip of the phage particles. After infecting, *Escherichia coli* TG1 cells were grown for 24 h and centrifuged at 2200 × *g* for 30 min to collect virus particles. Panx1-targeting nanobodies were retrieved from the resulting library of phage displayed nanobodies by biopanning on DUBCA cells. Selection rounds on DUBCA WT and DUBCA mPanx1 cells allowed to identify single individual colonies. These colonies were screened through an enzyme-linked immunosorbent assay on DUBCA mPanx1 cells for antigen recognition. Positive clones were selected and sequenced.

### Production of Panx1-targeting nanobodies

A total of 3 Panx1 nanobody clones (Nb1, Nb3 and Nb9) and the non-targeting Nb were selected for large-scale production. Then non-targeting Nb R3b23 is a nanobody targeting 5T2 multiple myeloma cell-produced M-proteins [18]. and is used as a negative control nanobody in this study. pMECS vectors containing the nanobody coding sequences were used to transform electrocompetent *Escherichia coli* WK6 cells. Single colonies of transformed *Escherichia coli* WK6 cells were grown at 37 °C until OD_600nm_ values of 0.6–0.9 were reached. For expression of the nanobody, an isopropyl-β-D-thiogalactoside (IPTG)-stock solution (Duchefa Biochemie) was added to a final concentration of 1 mM IPTG. After overnight incubation at 28 °C, cells were harvested and pelleted through centrifugation at 11,300 × *g* for 8 min. Then, periplasmic extracts were collected via an osmotic shock. Cell pellets were resuspended in a TES buffer, containing 500 mM sucrose (Duchefa Biochemie), 200 mM Tris–HCl (Merck) and 0.50 mM EDTA (Duchefa Biochemie) in water, and placed on ice for 6 h while shaking. The resuspended cell pellet was topped up with a double volume of 25% v/v diluted TES buffer and placed on ice while shaking. The next day, WK6 cells were centrifuged at 11,300 × *g* for 30 min and supernatant was collected. After repeating the osmotic shock procedure, immobilized metal affinity chromatography (IMAC) was performed on collected supernatant to capture produced nanobodies from periplasmic extracts. Periplasmic extracts were loaded on a His-Select Nickel Affinity Gel (Merck) and drained by gravity. After washing the resin with 20 bed volumes PBS, nanobodies were retrieved with an elution solution, consisting of 0.5 M imidazole (Merck) in PBS. IMAC-eluted samples were further purified on an AKTAxpress chromatography system equipped with a HiLoad S75 (16/60) column (Cytiva, Belgium). Finally, the purified nanobody solutions were concentrated in PBS using Vivaspin 5000 MW PES centrifugal concentrators (Satorius, Belgium).

### Flow cytometry with Panx1-targeting nanobodies

DUBCA WT, DUBCA mPanx1 and DUBCA hPanx1 cells were harvested from culture flasks by dissociation with TrypLE. Following centrifugation at 1500 × *g* for 5 min, microcentrifuge tubes (Corning) were filled with DUBCA cell suspensions at a density of 10^5^ cells in 1 mL flow cytometry buffer, PBS enriched with 1% w/v BSA. Series of dilutions of Nb1, Nb3, Nb9 and non-targeting Nb (0–1000 nM) in flow cytometry buffer were prepared and transferred to DUBCA cell suspensions. DUBCA cells were incubated separately with various concentrations of Nb1, Nb3, Nb9 and non-targeting Nb for 1.5 h at 4 °C. Thereafter, cells were centrifugated at 1600 × *g* for 3 min. Supernatant was removed and cells were incubated with Alexa Fluor^®^ 488-conjugated hemagglutinin antibody (Clone 901509, BioLegend, USA) (Table 1). After 20 min, cells were centrifugated at 1600 × *g* for 3 min and washed with PBS. Following 3 times washing with PBS, nuclear counterstaining was performed with Hoechst solution. Binding of Panx1-targeting nanobodies was detected on Hoechst positive cells using an Attune NxT flow cytometer (Thermo Fisher Scientific). Obtained values were used to plot binding curves and to assess the equilibrium dissociation constant (K_d_) of the Panx1-targeting nanobodies.

### Panx1 channel activity assay

DUBCA hPanx1 cells were cultured at a density of 12,000 cells/well in 200 µL/well cell culture medium using a 96-well culture plate (Corning). The next day, cell culture medium was changed with a classic buffer, containing 137 mM NaCl, 2.68 mM KCl, 11.90 mM NaHCO_3_, 0.42 mM NaH_2_PO_4_.H_2_O, 1 mM MgCl_2_, 2 mM CaCl_2_.2H_2_O, 5 mM HEPES and 0.1% w/v glucose (Merck), for 30 min at 37 °C with a constant supply of 5% CO_2_. Panx1 channels were opened by switching to a buffer with increased potassium concentration, containing 22.93 mM NaCl, 5 mM KCl, 5.95 mM NaHCO_3_, 0.21 mM NaH_2_PO4.H_2_O, 1 mM MgCl_2_, 2 mM CaCl_2_.2H_2_O, 5 mM HEPES and 0.1% w/v glucose. Stock solutions of carbenoxolone disodium salt (100 µM) (Merck), lanthanum trichloride (100 µM) (Merck), ^10^Panx1 (300 µM) (Thermo Fisher Scientific), Nb1, Nb3, Nb9 and non-targeting Nb (0–10,000 nM) were prepared in buffer with and without increased potassium concentration. Supernatants from each well were aspirated and preconditioned with appropriate preheated buffer samples in a humidified 5% CO_2_ incubator at 37 °C for 15 min. Cells were subsequently exposed to preheated buffer with higher potassium concentration for another 30 min at 37 °C with a constant supply of 5% CO_2_. Extracellular ATP levels were assessed using an ATP Bioluminescent Assay Kit (213-579-1) (Merck). The amount of emitted light by the samples was immediately measured with a VICTOR3 Multilabel Plate Reader (PerkinElmer, USA). Extracellular ATP release was expressed as the percentage of ATP relative to the release level triggered by the buffer with increased potassium concentration.

### In vitro inflammation assay

RAW264.7 cells were cultured at a density of 10^6^ cells/well in 1 mL/well culture medium using a 24-well culture plate. The next day, cell culture media was removed and RAW264.7 cells were washed with Dulbecco’s modified Eagle’s medium. RAW264.7 cells were incubated with LPS-buffer, Dulbecco’s modified Eagle’s medium containing 1 µg/mL LPS (L4391) (Merck), in a humidified 5% CO_2_ incubator at 37 °C for 4 h. Thereafter, cells were topped up with brefeldin A-buffer, Dulbecco’s modified Eagle’s medium supplemented with 300 ng/mL brefeldin A (Abcam, UK), and placed at 37 °C with a constant supply of 5% CO_2_ for 3 h. Stock solutions of Nb1, Nb3, Nb9 and non-targeting Nb (1000 nM) were prepared in Dulbecco’s modified Eagle’s medium and added to the appropriate wells. The anti-inflammatory effects of Panx1-targeting nanobodies were tested by treating RAW264.7 cells with a stock solution of 1000 nM nanobody solution, which is the quantity of nanobody that is commonly applied in in vitro functionality assays [19–22]. After an incubation period of 1 h, RAW264.7 cells were taken out the incubator and topped up with ATP-buffer, Dulbecco’s modified Eagle’s medium containing 5 mM ATP (Thermo Fisher Scientific), for a final incubation step of 30 min at 37 °C with a constant supply of 5% CO_2_. Next, supernatant was removed and cells were fixed with 4% w/v paraformaldehyde in PBS for 15 min at room temperature. RAW264.7 cells were washed 3 times with PBS and incubated with permeabilization buffer, PBS enriched with 0.1% v/v Triton X-100, for 10 min at room temperature. Subsequent blocking of the cells was performed with blocking buffer, permeabilization buffer containing 0.75% w/v glycine and 2% w/v BSA. Cells were blocked for 15 min at room temperature followed by incubation with a primary antibody directed against IL-1β (Clone ab254360, Abcam) (Table 1). Afterwards, cells were washed 3 times with PBS and incubated with an Alexa Fluor^®^ 488-conjugated secondary antibody goat anti-rabbit (Clone ab150077, Abcam) (Table 1). After 3 times washing with PBS, nuclear counterstaining was performed with Hoechst solution. Detection of IL-1β was carried out on Hoechst positive cells using an Attune NxT flow cytometer. For positive controls, RAW264.7 cells were preincubated for 1 h with a stock solution of dexamethasone (100 µM) (Merck) in Dulbecco’s modified Eagle’s medium at 37 °C with a constant supply of 5% CO_2_.

### Radiolabeling of Panx1-targeting nanobodies

Panx1 and non-targeting nanobody coding sequences were recloned into a pHEN6c vector for Nb production. For the labeling, Technetium-99m (^99m^Tc)-tricarbonyl precursor was prepared by adding 1.5 mL of ^99m^Tc-eluate from a ^99m^Mo/^99m^Tc generator (740 MBq-3.7 GBq) (Drytec, UK) to a lyophilisation kit (IsoLink, Netherlands). After placing the kit in a boiling water bath for 20 min, 50 µg nanobody was mixed with ^99m^Tc-tricarbonyl precursor and incubated for 1.5 h at 50 °C. Thereafter, radiolabeled nanobodies were purified from unbound ^99m^Tc-tricarbonyl precursor and aggregates by filtration over a NAP-5 column (Cytiva) and a 0.22 µm membrane filter (Merck), respectively. Before injecting the nanobodies, radiochemical purity of labeled nanobodies was measured with instant thin-layer chromatography using silica gel impregnated glass fiber sheets (Pall Life Sciences, Belgium). Nanobodies showing high radiochemical purity, *i.e.* at least 98%, were used for in vivo biodistribution studies.

### In vivo biodistribution of Panx1-targeting nanobodies

Healthy adult male C57BL/6 J mice were intravenously injected with 5 µg of radiolabeled nanobody. One hour post injection, mice were anesthetized with 75 mg/kg ketamine and 1 mg/kg medetomidine (Ketamidor^®^) (Richter Pharma AG, Austria) via intraperitoneal injection and SPECT/CT imaging was performed using a Vector^+^ scanner (MILabs, Netherlands). Imaging set-up consisted of a 1.5 mm 75-pinhole general purpose collimator, in spiral mode with 6 bed positions. Total SPECT scanning time was 15 min with 150 s per position and CT scanning (60 kV and 615 mA) was 2 min. Following imaging, mice were euthanized and organs were collected and weighed. Radioactivity in each organ was determined using a Wizard^2^ γ-counter (PerkinElmer). Uptake in each organ was corrected for radioactivity decay and calculated as percentage of injected activity per gram of organ. SPECT/CT image analysis was performed using AMIDE (UCLA, USA) and Osirix (Pixmeo, Switzerland) software.

### Acute liver injury mouse model

Mice were starved 12–14 h *prior* to APAP administration. APAP (Merck) was dissolved in PBS, heated to 37 °C and injected intraperitoneally at 300 mg/kg body weight. The control group was not injected with APAP. After 2 h, APAP-overdosed mice was additionally administered either Nb1, Nb3, Nb9 or non-targeting Nb diluted in PBS at 10 mg/kg body weight or with *N*-acetylcysteine (NAC) (Merck) in PBS at 200 mg/kg body weight through intraperitoneal injection. All mice were anesthetized with 75 mg/kg ketamine (Nimatek^®^) (Dechra, UK) and 10 mg/kg xylazine (Rompun^®^) (Bayer, Germany) via intraperitoneal injection and euthanised 24 h following APAP overdosing. The methodology for in vivo dose calculation was adopted from previous works [23, 24] and resulted in a dose of 10 mg/kg Panx1-targeting nanobody, thereby assuming 100% distribution in the blood volume that is approximately 8% of the body weight. Consequently, the selected dose of 10 mg/kg corresponds to the 7,500 nM nanobody in vitro concentration, which in turn led to *ca.* 50% inhibition of Panx1 channel activity in vitro. Blood, collected by cardiac puncture, was centrifugated at 2000 × *g* for 10 min, and serum was stored at − 80 °C. Livers were excised and fragments were fixed in 4% phosphate-buffered formalin (ProSan, Belgium) for 6 h at 4 °C or snap-frozen in liquid nitrogen for 15 min with storage at − 80 °C.

### Immunoblot analysis of liver proteins

Flash frozen liver tissue was homogenised in RIPA buffer supplemented with 1% v/v EDTA and 1% v/v protease and phosphatase inhibitor cocktail by mixing the samples for 20 s with an Ultra-Turrax^®.^ T25 Disperser (IKA, Belgium). Following homogenization, samples were centrifugated at 14,000 × *g* for 20 min and protein concentrations were determined by means of a BCA assay. Electrophoresis, blotting and blocking of membranes were performed as described above. Next, membranes were incubated with primary antibody directed against caspase-1 (Clone sc-514, Santa Cruz, USA), CYP2E1 (Clone HPA009128, Atlas Antibodies, Sweden), IL-1β (Clone ab254360), NLRP3 (Clone ab263899, Abcam) and Panx1 (Clone D9M1C) (Table 1). After 3 times washing with TBS/T, membranes were subsequently incubated with horseradish peroxidase-conjugated secondary antibodies (Clone P0447 and Clone P0448, Dako) (Table 1). Membranes were washed 3 times with TBS/T and detection of liver protein expression was carried out by means of an enhanced chemiluminescence Western Blotting Substrate Kit and a ChemiDoc MP Imaging System. For semi-quantification purposes, liver protein expression levels were normalised against total protein loading using Image Lab software.

### Serum cytokine analysis

Serum cytokine levels were measured using a mouse inflammation antibody array according to the providers’ instructions (ab133999) (Abcam). After blocking the antibody array membranes with the provided blocking buffer for 30 min, membranes were incubated with collected serum samples for 24 h. Thereafter, membranes were washed with provided washing buffers for 25 min and treated with the supplied cocktail of biotin-conjugated antibodies. After 2 h, antibody array membranes were washed for another 25 min. Serum cytokine levels were determined by incubating membranes with the provided horseradish peroxidase-conjugated streptavidin solution for 2 h and chemiluminescence detection solution for 2 min. Chemiluminescence signals were measured with a ChemiDoc MP Imaging System and densitometric analysis was performed with Image Lab Software according to the manufacturer’s instructions. Upon background subtraction and normalisation to the amount of biotin-conjugated IgG protein printed on each membrane, cytokine levels of 40 inflammatory factors were calculated and expressed as relative alterations compared to APAP-overdosed mice.

### Evaluation of liver tissue

For microscopic evaluation, formalin-fixed liver tissues were placed into histology cassettes (VWR International, Belgium). Liver tissue was subsequently submerged in a series of 90%, 95% and 100% v/v ethanol (Merck) solutions for 2 h. After exchanging ethanol with xylene (Chem-Lab, Belgium) 3 times for 2 h, liver tissue was embedded in paraffin (Prosan) at 60 °C. Tissue sections of 10 µm were cut with a SM2010 R sliding microtome (Leica, Belgium) and placed on microscope adhesive glass slides (VWR International). To ensure a clear representation of the treatments, 3 tissue sections per liver sample, separated by at least 100 µm, were used. Thereafter, liver tissue sections were deparaffinized in xylene for 30 min, rehydrated in ethanol by washing the slides in a series of 100%, 90% and 70% v/v ethanol solutions for 1 min and washed in PBS for 5 min. Tissue samples were stained with hematoxylin and periodic acid Schiff base (H-PAS) by treating the samples subsequently with Schiff’s reagent (Merck) and hematoxylin (ProSan) for 5 min. Histological evaluation was carried out with an Olympus IX 81 bright field microscope (Olympus, Belgium) and liver samples were blindly analysed using ToupView software (ToupTek Photonics, China). The percentage of necrosis was estimated by measuring the necrotic area of microscopic fields compared to the cross-sectional areas over the entire section. Liver histopathology was evaluated by using Suzuki’s score-method quantifying for congestion, vacuolization and necrosis on stained sections (Table 2).

**Table 2:** Suzuki’s score-method

Suzuki Score			
Score	Congestion	Vacuolization	Necrosis
0	None	None	None
1	Minimal	Minimal	Single cell necrosis
2	Mild	Mild	± 30%
3	Moderate	Moderate	± 60%
4	Severe	Severe	> 60%

Liver histopathology was evaluated by using Suzuki’s score-method quantifying for congestion, vacuolization and necrosis on hematoxylin and periodic acid Schiff base (H-PAS)-stained sections

### Statistical analysis

All data were analysed using GraphPad Prism 7 software (GraphPad Software Inc., USA) and are presented as means ± standard deviation (S.D.).

## Results

### Generation of cross-reactive Panx1-targeting nanobodies

**Fig. 1:**
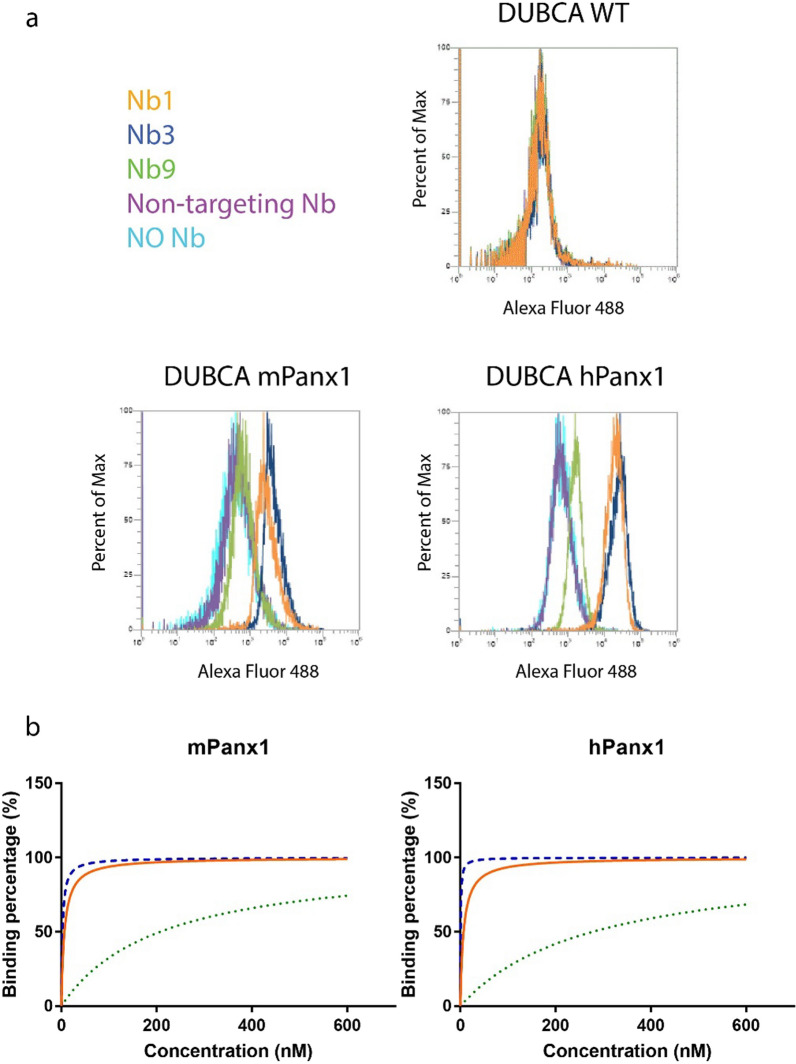
Panx1-targeting nanobodies show cross-reactive binding to murine and human Panx1. **a** Flow cytometry analysis of the binding of Panx1-targeting nanobodies (Nb1, Nb3 and Nb9) and non-targeting Nb (1000 nM) to DUBCA wild-type (WT) cells and DUBCA cells overexpressing mouse Panx1 (mPanx1) or human Panx1 (hPanx1). Representative data of 3 independent experiments is shown. **b** Flow cytometry analysis of the binding of different concentrations of Panx1-targeting nanobodies (Nb1, Nb3 and Nb9) (0–600 nM) to DUBCA mPanx1 and hPanx1 cells. Binding values for Nb1 (orange), Nb3 (blue) and Nb9 (green) were plotted to define affinity towards mPanx1 and hPanx1 (n = 3 independent experiments). Data were expressed as means

To generate Panx1-targeting nanobodies, a llama was immunised with a DNA vector encoding for mPanx1 and subsequently boosted with DUBCA cells overexpressing mPanx1. Next, cDNA from peripheral blood lymphocytes of the immunised llama was used to construct an immune nanobody library. Subsequently, Panx1-targeting nanobodies were retrieved via phage display and biopannings on mPanx1 overexpressing DUBCA cells (Additional file 1: Fig. S1 and Additional file 1: Fig. S2). Screening and sequencing resulted in the identification of 7 different nanobody families, *i.e.* group of nanobodies with a high similarity in their 3^rd^ complementary-determining region sequence. Representative clones of each family were selected for further characterisation. As such, 3 Panx1-targeting nanobodies were identified by flow cytometry to cross-react with mPanx1 and hPanx1 (Nb1, Nb3 and Nb9).  mPanx1 and hPanx1 protein only differ by 6 amino acids in the extracellular loop regions (The UniProt Consortium). Indeed, the 3 identified Panx1-targeting nanobodies bound to both DUBCA mPanx1 and DUBCA hPanx1 cells but not untransduced DUBCA cells, while no binding was observed for a non-targeting Nb (Fig. 1a). Affinity for mPanx1 and hPanx1 was further evidenced by incubating DUBCA WT, DUBCA mPanx1 and DUBCA hPanx1 cells with individual Panx1 nanobody clones in different concentrations ranging from 0 up to 600 nM (Fig. 1b). Recorded binding values were used to plot binding capacity curves and to define K_d_ values. K_d_ values of 6.6 nM, 2.4 nM and 206.6 nM for mPanx1 and 6.9 nM, 0.7 nM and 277.4 nM for hPanx1 were measured for Nb1, Nb3 and Nb9, respectively (Fig. 1b and Table 3). Thus, all 3 identified Panx1-targeting nanobodies are able to bind Panx1 proteins and show similar affinities to mPanx1 and hPanx1.

**Table 3:** Equilibrium dissociation constants of Panx1-targeting nanobodies

Equilibrium dissociation constants (K_d_) of Panx1-targeting nanobodies		
Nanobody	mPanx1	hPanx1
Nb1	6.6 ± 3.0 nM	6.9 ± 2.9 nM
Nb3	2.4 ± 1.5 nM	0.7 ± 2.5 nM
Nb9	206.6 ± 71.7 nM	277.4 ± 67.2 nM

Affinity for mouse Panx1 (mPanx1) and human Panx1 (hPanx1) was evaluated by incubating DUBCA WT, DUBCA mPanx1 and DUBCA hPanx1 cells with individual Panx1 nanobody clones in different concentrations ranging from 0 up to 600 nM. Recorded binding values were used to define equilibrium dissociation constant (K_d_) values

### Panx1-targeting nanobodies block Panx1 channel activity and show anti-inflammatory effects in vitro

**Fig. 2:**
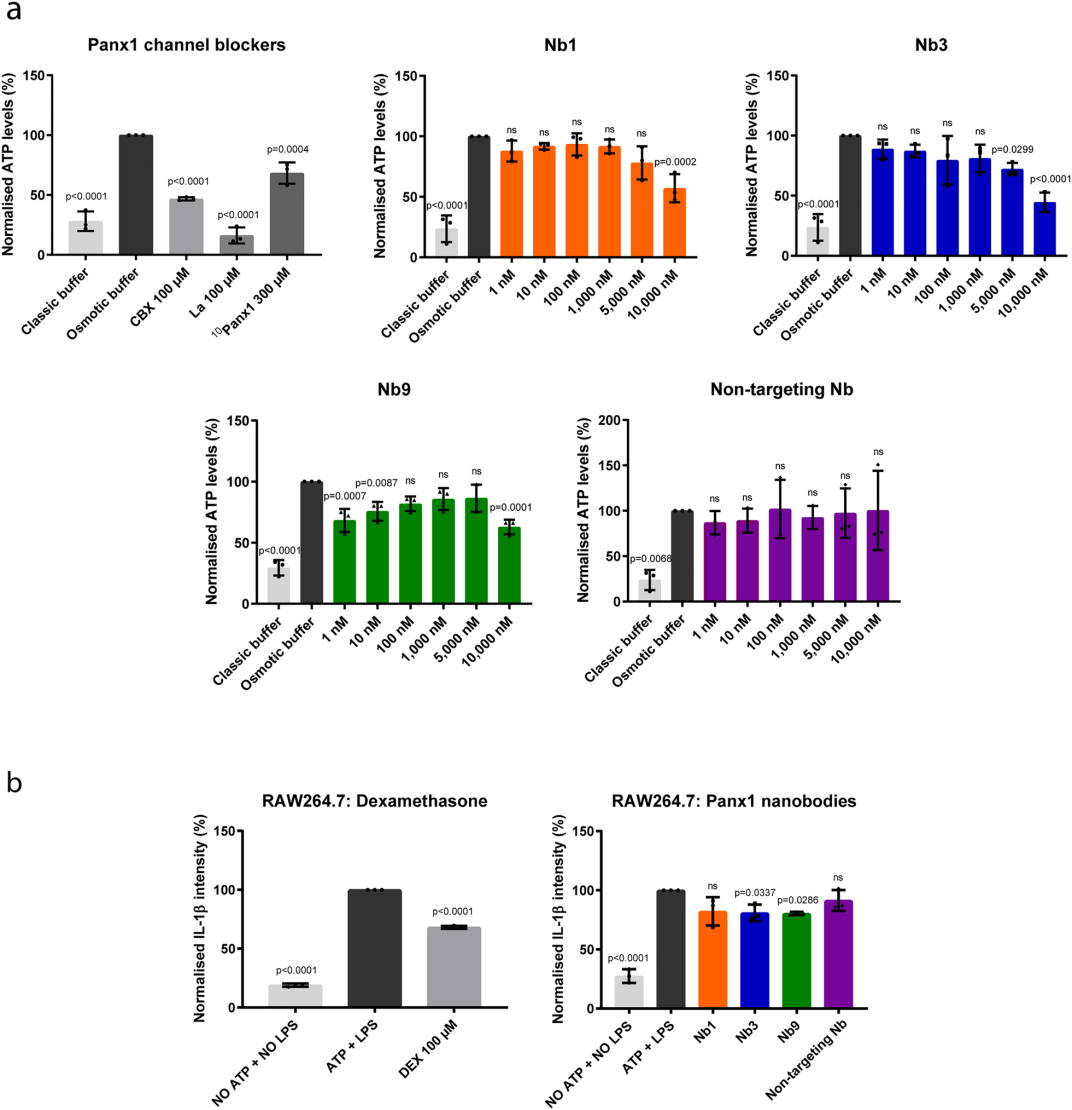
Panx1-targeting nanobodies block Panx1 channel activity and show anti-inflammatory effects in vitro. **a** Panx1-targeting nanobodies (Nb1, Nb3 and Nb9) inhibit human Panx1 (hPanx1)-mediated extracellular release of ATP following potassium-induced channel opening in hPanx1-overexpressing DUBCA cells. Blocking effects of Nb1, Nb3, Nb9, non-targeting Nb (0–10,000 nM), carbenoxolone (CBX) (100 µM), lanthanum (La) (100 µM) and ^10^Panx1 (300 µM) were measured and expressed as the percentage of ATP relative to the release level triggered by potassium-enriched buffer (osmotic buffer) (n = 3 independent experiments). **b** Anti-inflammatory effects of Panx1-targeting nanobodies (Nb1, Nb3 and Nb9) were measured by analysing IL-1β signals in RAW264.7 cells. Anti-inflammatory effects of Nb1, Nb3, Nb9, non-targeting Nb (1000 nM) and dexamethasone (DEX) (100 µM) were measured and expressed as the percentage of IL-1β relative to the level triggered by LPS + ATP (n = 3 independent experiments). All data was analysed by parametric 1-way analysis of variance followed by post hoc tests with Bonferroni’s correction. Data were expressed as means ± S.D

The Panx1 channel inhibitory effects of the Panx1-targeting nanobodies were assessed by measurement of hPanx1-mediated extracellular release of ATP following potassium-induced channel opening in hPanx1-overexpressing DUBCA cells. Established Panx1 channel inhibitors such as carbenoxolone, lanthanum and ^10^Panx1, showed significantly reducted extracellular ATP levels, reflecting inhibition of Panx1 channel activity (Fig. 2a) [16, 17]. Panx1-targeting nanobodies along with the non-targeting Nb were evaluated in this experimental set-up in concentrations ranging from 0 to 10,000 nM. Unlike for the non-targeting Nb, decreased ATP levels were noticed following exposure to Nb1, Nb3 and Nb9 (Fig. 2a). Besides Panx1 channel blocking ability, the anti-inflammatory potential of Panx1-targeting nanobodies was tested by measurement of IL-1β levels in a LPS/ATP-mediated inflammation model using RAW264.7 cells. Focus was put on IL-1β as this prototypical pro-inflammatory cytokine has been identified as one of the main cytokines of acute inflammation and NLRP3 inflammasome signaling, a process in which Panx1 channels have been shown to play an essential role [17, 25–27]. LPS and ATP were used to evoke inflammatory effects in vitro, representing the increased IL-1β-signal (Fig. 2b). The anti-inflammatory and immunosuppressant drug dexamethasone was used as positive control, and indeed decreased IL-1β intensity in RAW264.7 cells (Fig. 2b). Nb3 and Nb9 possess anti-inflammatory potential as evidenced by lowered IL-1β signals in RAW264.7 cells (Fig. 2b). Nb1 and the non-targeting Nb had no effect on IL-1β signals. Altogether, 2 of the identified Panx1-targeting nanobodies are able to block Panx1 channel activity and show anti-inflammatory effects in vitro.

### Panx1-targeting nanobodies show anti-inflammatory effects in vivo

**Fig. 3:**
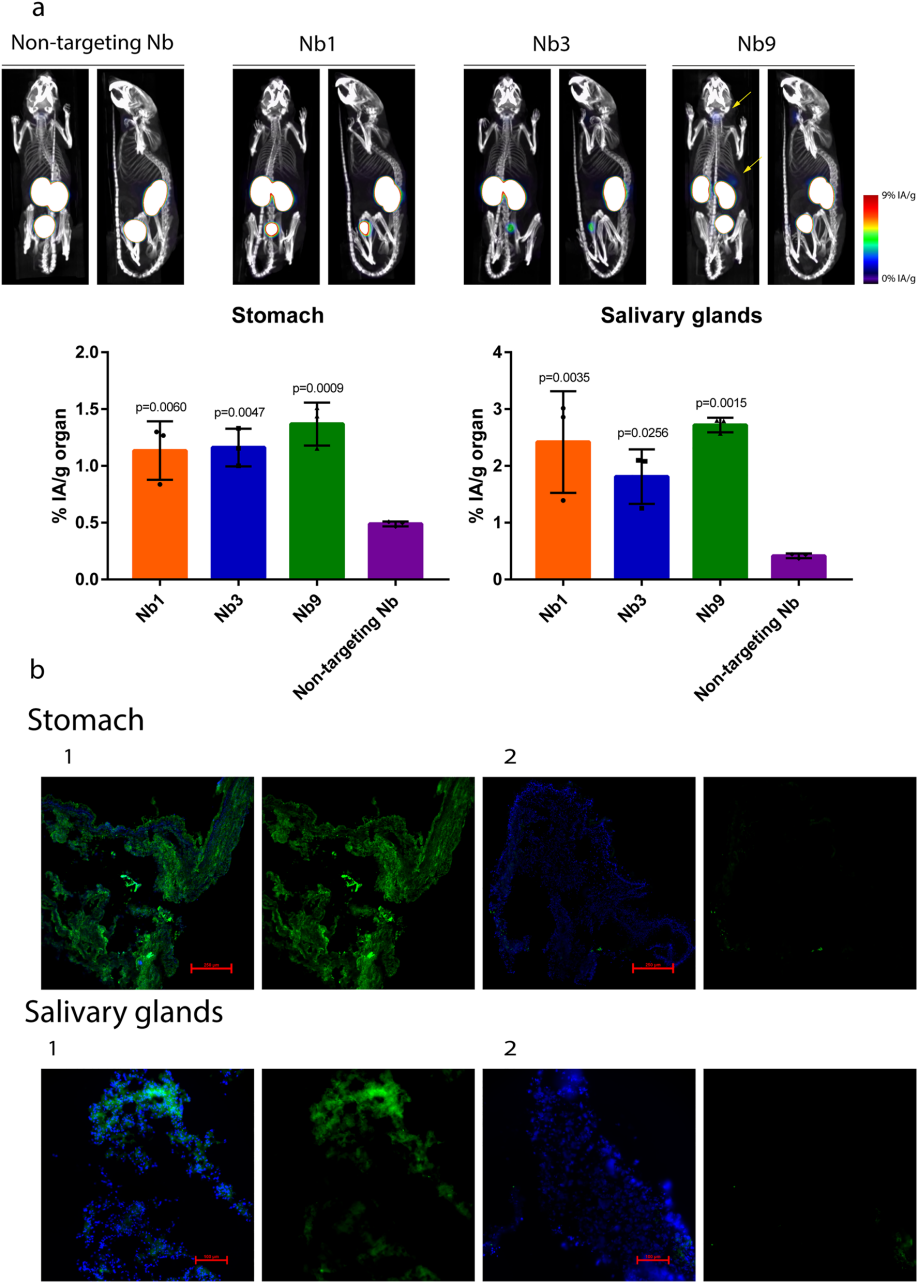
In vivo biodistribution of Panx1-targeting nanobodies. **a** Nb1, Nb3, Nb9 and non-targeting Nb were radiolabeled with Technetium-99m (^99m^Tc), and 5 µg of radiolabeled nanobody was injected intravenously in healthy adult mice. SPECT/CT imaging was performed 1 h after injection. Biodistribution of nanobodies was determined by γ-counting of stomach and salivary glands and expressed as percentage of injected activity per gram of organ (n = 3 animals per group). All data was analysed by parametric 1-way analysis of variance followed by post hoc tests with Bonferroni’s correction. Data were expressed as means ± S.D. **b** Stomach and salivary gland sections were subjected to (1) immunohistochemistry analysis of Panx1 (green) with nuclear counterstaining (blue). For (2) negative controls, the primary antibody directed against Panx1 was omitted. Scale bars represent 100 or 250 µm (red). Representative data of 3 independent experiments

We next evaluated the in vivo applicability of the Panx1-targeting nanobodies. First, in vivo biodistribution of Panx1-targeting nanobodies was determined via SPECT/CT imaging. To this end, the Panx1-targeting nanobodies were radiolabeled with ^99m^Tc and injected intravenously in healthy adult mice. One hour post-injection, SPECT/CT imaging and subsequent γ-counting of isolated organs revealed low uptake of ^99m^Tc-labeled nanobodies in nearly all organs, except for the kidney and bladder, which is the known secretion route of nanobodies (Fig. 3a and Additional file 1: Fig. S3) [28]. Albeit low, but significant, an increased uptake was seen for Panx1-targeting nanobodies in salivary glands and stomach compared to the non-targeting Nb (Fig. 3a). Expression of Panx1 proteins in salivary glands and stomach was confirmed by immunohistochemistry analysis (Fig. 3b).

**Fig. 4:**
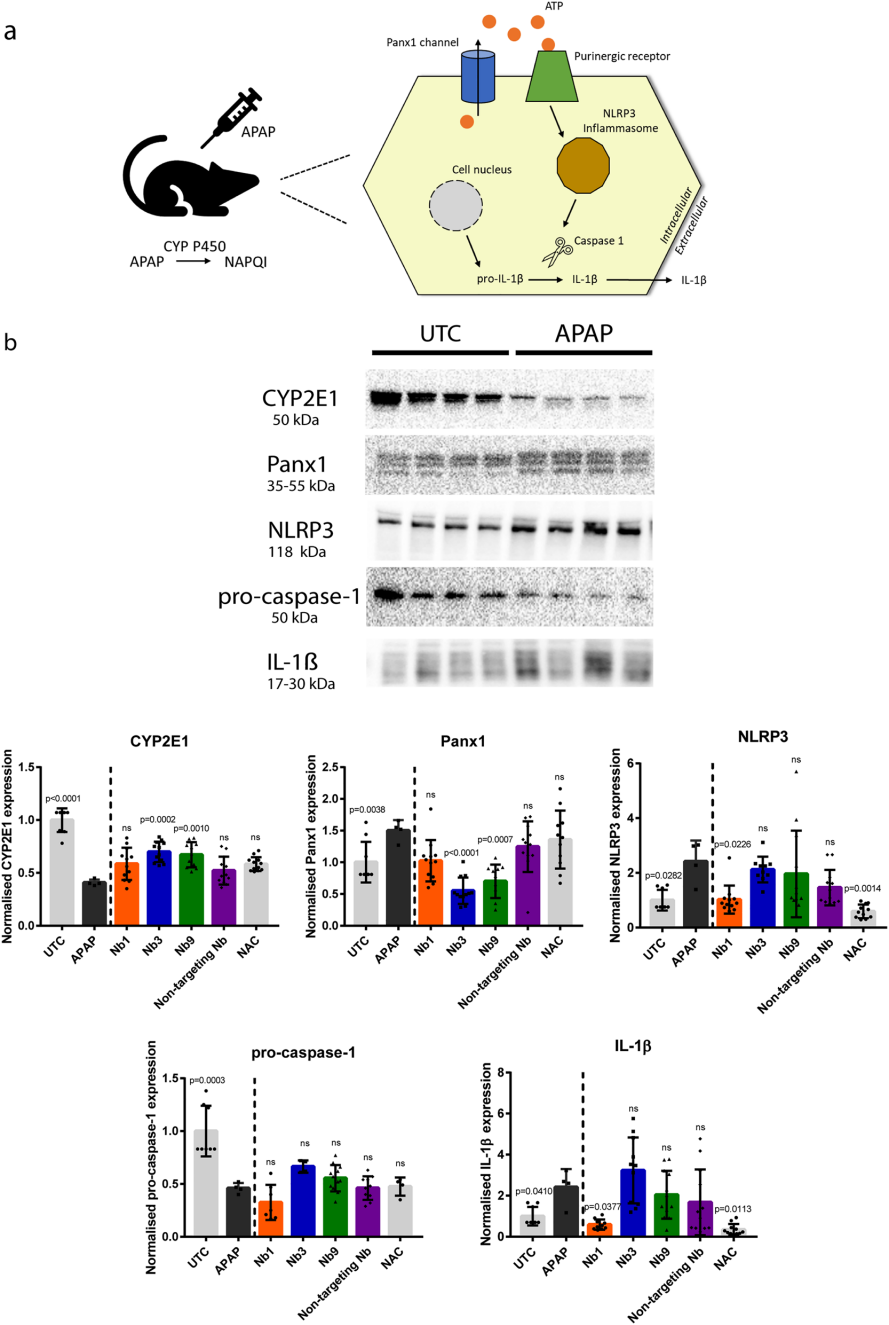
Panx1-targeting nanobodies interact with the NLRP3 inflammatory cascade in acetaminophen-overdosed mice. **a** Overview of the NLRP3 signaling cascade in acetaminophen (APAP)-induced hepatotoxicity. Overdosing mice with APAP triggers oxidative metabolization catalyzed by cytochrome (CYP) P450 enzymes in hepatocytes. Associated-NAPQI formation causes necrosis, which triggers a Panx1-mediated inflammatory response in the liver. The opening of Panx1 channels leads to extracellular ATP release underlying NLRP3-mediated maturation of IL-1β. **b** Adult mice were overdosed with APAP (300 mg/kg) or kept untreated (UTC). After 2 h, some mice were additionally administered either nanobody (Nb1, Nb3, Nb9 or non-targeting Nb (10 mg/kg)) or *N*-acetylcysteine (NAC) (200 mg/kg). Sampling was performed 24 h after APAP overdosing. Hepatic protein levels of CYP2E1, Panx1, NLRP3, pro-caspase-1 and IL-1β were assessed by immunoblot analysis. Protein levels were normalised against the total protein content and expressed as relative alteration compared to APAP mice (n = 4 (UTC and APAP) or n = 12 (Nb1, Nb3, Nb9, non-targeting Nb and NAC) animals per group). All data was analysed by unpaired t-tests with Welch’s correction or parametric 1-way analysis of variance followed by post hoc tests with Bonferroni’s correction. Data were expressed as means ± S.D

**Fig. 5:**
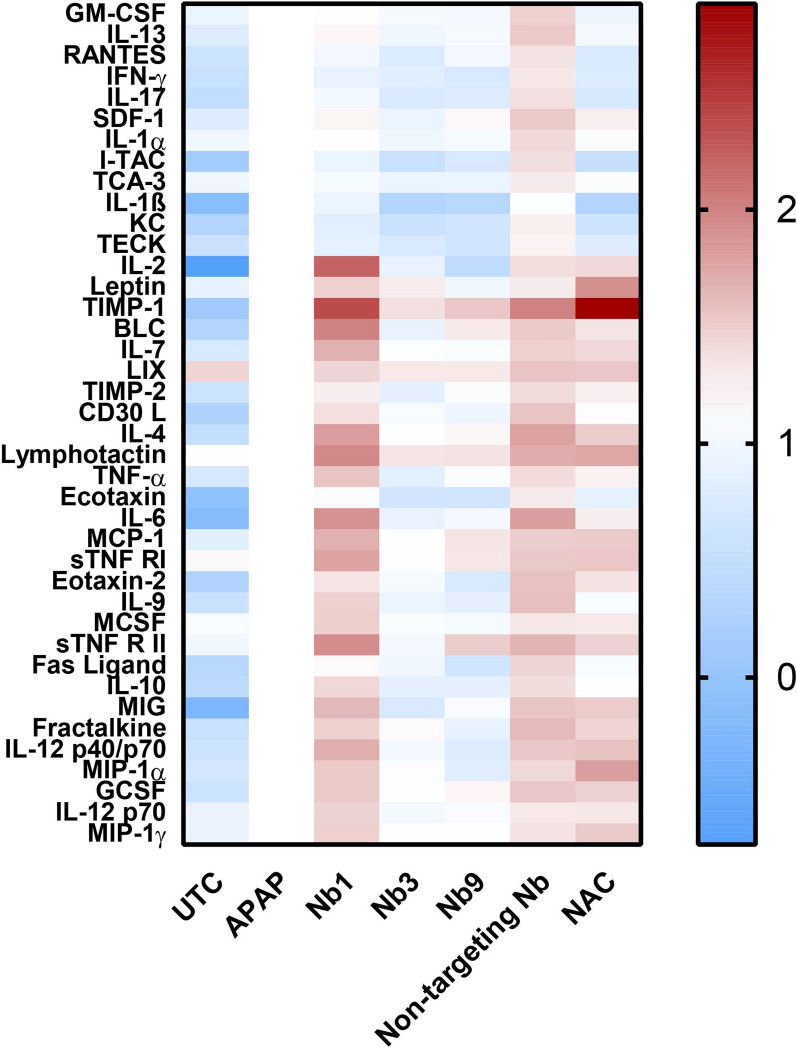
Panx1-targeting nanobodies reduce the serum levels of inflammatory cytokines in acetaminophen-overdosed mice. Adult mice were overdosed with acetaminophen (APAP) (300 mg/kg) or kept untreated (UTC). After 2 h, some mice were additionally administered either nanobody (Nb1, Nb3, Nb9 or non-targeting Nb (10 mg/kg) or *N*-acetylcysteine (NAC) (200 mg/kg). Sampling was performed 24 h after APAP overdosing. Serum cytokine levels were measured using chemiluminescence detection and densitometric analysis. Cytokine levels were normalised and expressed as relative alterations compared to APAP mice. Data are presented in a heat map with a colour scale highlighting low values in blue and high values in red (n = 3 independent experiments)

**Fig. 6:**
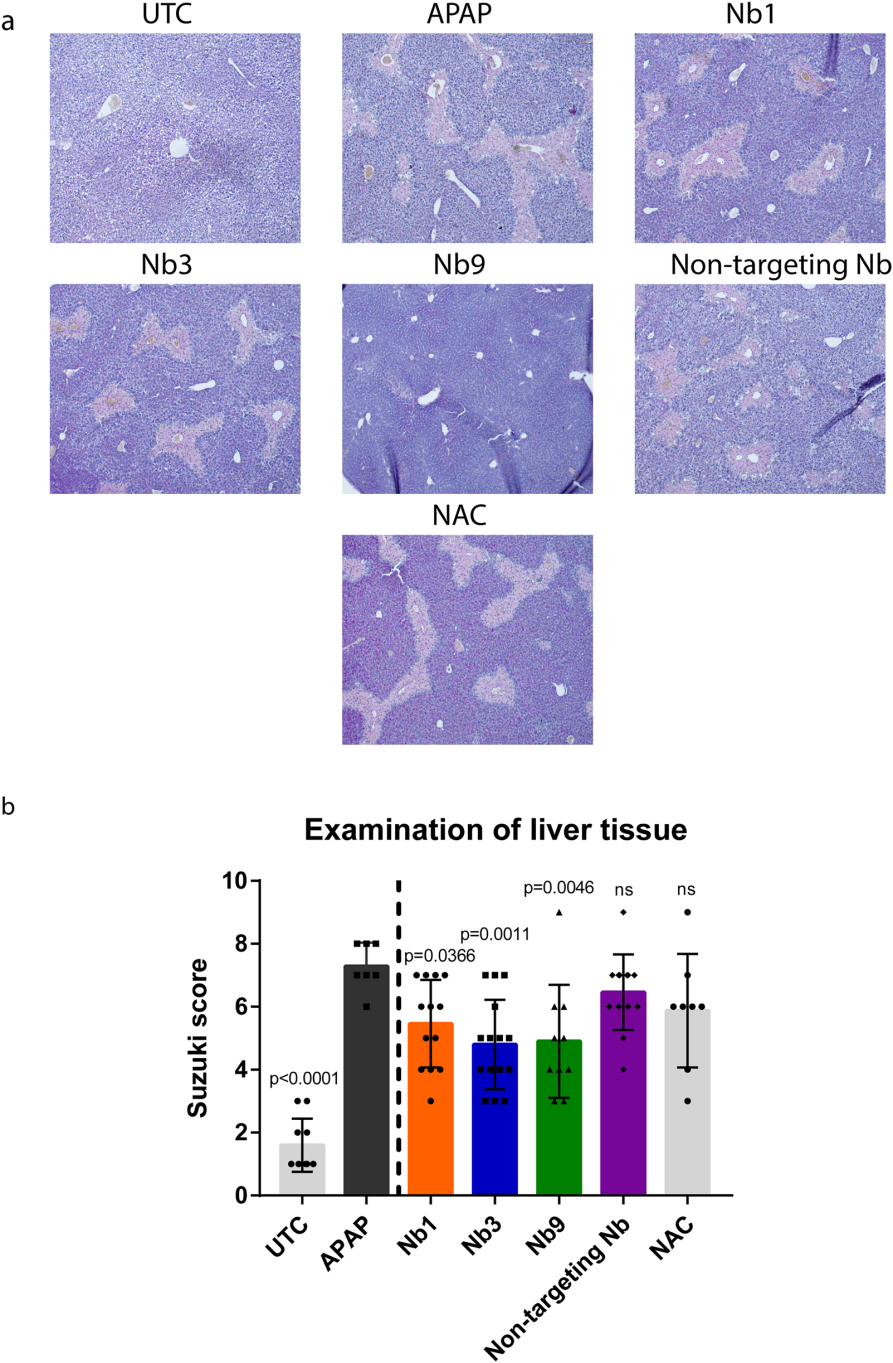
Panx1-targeting nanobodies reduce liver histopathology in acetaminophen-overdosed mice. Adult mice were overdosed with acetaminophen (APAP) (300 mg/kg) or kept untreated (UTC). After 2 h, some mice were additionally administered either nanobody (Nb1, Nb3, Nb9 or non-targeting Nb (10 mg/kg)) or *N*-acetylcysteine (NAC) (200 mg/kg). Sampling was performed 24 h after APAP overdosing. **a** Liver sections (10 µm) were stained with hematoxylin and periodic acid Schiff base (H-PAS) and **b** liver histopathology was evaluated using Suzuki’s score-method quantifying for congestion, vacuolization and necrosis (n = 4 (UTC and APAP) or n = 12 (Nb1, Nb3, Nb9, non-targeting Nb and NAC) animals per group). All data was analysed by parametric 1-way analysis of variance followed by post hoc tests with Bonferroni’s correction. Data were expressed as means ± S.D

We thereafter set out to investigate the anti-inflammatory potential of the Panx1-targeting nanobodies in vivo using a human-relevant mouse model of acute liver injury. This model is based on overdosing mice with APAP. In overdose, normal APAP metabolism is disturbed and APAP undergoes oxidative metabolization catalyzed by cytochrome (CYP) P450 enzymes in hepatocytes (Fig. 4a) [5]. Associated-NAPQI formation causes necrosis, which triggers a Panx1-mediated inflammatory response in the liver [9, 10]. An overdose of APAP was used to trigger NLRP3 inflammasome activation in vivo [29], a pathway in which Panx1 proteins are identified as essential players [26, 30]. The opening of Panx1 channels indeed underlies NLRP3-mediated maturation of IL-1β (Fig. 4a) [26, 30]. Accordingly, increased quantities of Panx1, NLRP3 and IL-1β proteins were measured compared to untreated control (UTC) animals (Fig. 4b). Moreover, APAP-induced inflammation was underscored by lower liver levels of CYP P450 2E1 and pro-caspase-1 (Fig. 4b). In order to investigate the anti-inflammatory effects of Panx1-targeting nanobodies in mice, nanobodies were injected 2 h after APAP-injection. Another group of mice was treated with NAC, the only clinically approved antidote for APAP-induced liver injury [5]. NAC was found to reduce serum levels of merely a subset of inflammatory cytokines, including IL-1β, in APAP-overdosed mice. Other cytokines, like IL-6, IL-10 and MCP-1, were unaffected (Fig. 5). While treatment with the non-targeting Nb did not lower the serum levels of any cytokine (Fig. 5), the Panx1-targeting nanobodies affected several of the cytokines under investigation. Interestingly, Nb1 treatment displayed similar results as NAC treatment, whereas treatment with Nb3 and Nb9 resulted in reduced serum levels of most measured inflammatory cytokines indicating a more comprehensive anti-inflammatory response compared to NAC (Fig. 5). These prominent anti-inflammatory responses were accompanied by a decrease in serum levels of IL-10. IL-10 is often considered as a potent anti-inflammatory cytokine [31, 32]. This cytokine plays a critical role in the regulatory phase of inflammation attenuation [33, 34]. Consistent with this, our IL-10-data show that there was a less severe inflammatory response in liver following treatment of APAP-overdosed mice with Nb3 and Nb9. In addition to serum cytokines, we also assessed the effects of nanobody treatment on expression levels of different hepatic proteins. Application of the non-targeting Nb had no effect on CYP2E1, Panx1, NLRP3, pro-caspase-1 and IL-1β levels. By contrast, treatment with Nb3 and Nb9 was able to partially restore CYP2E1 protein expression. Administration of Nb3 and Nb9 to the mice had also an effect on hepatic Panx1 proteins as shown by lower expression levels. On the other hand, Nb1 as well as NAC lowered NLRP3 and IL-1β quantities. None of the Panx1-targeting nanobodies or NAC were found to affect pro-caspase-1 levels (Fig. 4b). Furthermore, liver tissue was evaluated following treatment of APAP-overdosed mice with Panx1-targeting nanobodies. H-PAS-stained liver sections were examined microscopically (Fig. 6a) and evaluated by using Suzuki’s score-method (Fig. 6b). Application of the non-targeting Nb and NAC had no effect. Treatment with Nb1, Nb3 and Nb9 reduced the Suzuki score (Fig. 6b). Moreover, protective effects of Panx1-targeting nanobodies can be linked to improvement on congestion and vacuolization. The lower Suzuki scores were, however, not accompanied with changes in necrotic cell death (Additional file 1: Fig. S4). Collectively, these data indicate that the identified Panx1-targeting nanobodies reduce inflammation in a mouse model of acute liver injury.

## Discussion

Although Panx1 proteins were only discovered in 2000, there is a large body of evidence showing that Panx1 channels play an important role in various physiological processes [11, 35, 36]. However, Panx1 channels have yet been mainly studied in a pathological context. Indeed, Panx1 channel-based communication is considered as a key event in inflammation. Specifically, the opening of Panx1 channels stimulates NLRP3 inflammasome signaling to activate IL-1β production. Such pro-inflammatory role of Panx1 channels is also seen during immune cell activation [13]. Opening of Panx1 channels is implicated in a wide array of pathologies, including airway inflammation, autoimmune encephalomyelitis, brain trauma injury, joint pain, liver disease and multiple sclerosis [37–42]. We suggest a role for Panx1 channels as a generic drug target to treat inflammation. Unfortunately, clinical exploration and translation is largely hampered by the ubiquitous lack of specific and/or in vivo-applicable inhibitors [43]. Since Panx1 proteins share structural properties with other cell plasma membrane transport proteins, the identification of specific Panx1-binding drugs remains a challenging endeavor. The present work represents a breakthrough in this regard by pioneering the generation of nanobody-based inhibitors of Panx1 channels.

Following immunization of a llama, we were able to identify 3 different nanobodies that showed affinity for both human and murine Panx1 in the nM range. Despite Nb9 having a lower affinity for Panx1 compared to Nb1 and Nb3, all 3 Panx1-targeting nanobodies were found to modulate Panx1 channel activity in vitro. Moreover, it should be mentioned that the Panx1-targeting nanobodies achieved similar inhibitory effects on Panx1 channels as observed for ^10^Panx1-treated cells by using 30 times smaller concentrations. As a result, the Panx1-targeting nanobodies outperformed the Panx1-mimetic peptide ^10^Panx1, the gold standard Panx1 channel inhibitor, in terms of channel blocking capacity. This may be due to efficient antigen recognition. Indeed, nanobodies succeed in targeting epitopes selectively and with high affinity. The small size and convex paratope of nanobodies enable binding hidden and cryptic epitopes of cell plasma membrane surface-associated proteins [44, 45]. Therefore, the use of nanobodies might be advantageous over small molecules, peptides and monoclonal antibodies. Subsequently, the functional relevance of nanobody-mediated blocking effects was tested in vitro. Both Nb3 and Nb9 reduced IL-1β amounts in RAW264.7 cells. Despite the low K_d_ value, no anti-inflammatory effects were observed for Nb1. The affinity of Panx1-targeting nanobodies is thus not directly linked to their in vitro efficacy.

The in vivo-applicability of Panx1-targeting nanobodies was examined through biodistribution studies in mice. Consistent with the expectations, the Panx1-targeting nanobodies were found to exhibit typical nanobody in vivo features, including low uptake in organs and rapid clearance through kidney filtration [46, 47]. In addition, increased uptake was measured for the Panx1-targeting nanobodies in salivary glands and stomach compared to the non-targeting Nb. The accumulation of the Panx1-targeting nanobodies is most likely caused by the pronounced expression of Panx1 proteins in both tissues. While peptides such as ^10^Panx1 cope with stability issues (*i.e.* ^10^Panx1 has a half-life of *ca.* 2 min in human plasma [48]), nanobodies show robustness to proteolytic degradation, elevated pressure, chemical denaturants, temperature fluctuations and pH extremes [44, 45]. Moreover, the targeting of nanobodies can be improved by modifying the in vivo pharmacokinetics of nanobodies. In this regard, mutating amino acid residues to create more human-like and (thermo)stable molecules, PEGylation and PASylation of nanobodies, linking different nanobodies to fabricate greater constructs and fusing nanobodies to an Fc-chain or anti-albumin nanobody to create half-life extended nanobody formats are among acknowledged strategies to generate complex nanobody formats [49–57].

To further evaluate the therapeutic potential of the Panx1-targeting nanobodies, a human-relevant experimental model of hepatotoxicity related to acute liver injury, namely DILI induced by APAP overdosing, was used [5]. A single injection of 300 mg/kg APAP is known to cause hepatic damage in fasted mice [58]. This is accompanied by inflammasome activation, a process in which Panx1 channels have been shown to play an essential role. Our group previously showed that APAP overdosing is associated with increased Panx1 mRNA and protein expression as well as enhanced Panx1 channel activity [9, 10]. Furthermore, our group previously reported that whole-body Panx1 knock-out mice are partially protected against APAP-mediated injury. Although liver inflammatory markers were unaffected, the knockdown of Panx1 expression led to decreased bio-activation of APAP, less liver damage, lowered oxidative stress and differential expression levels of a number of genes linked to xenobiotic biotransformation, oxidative stress and inflammation upon APAP overdosing [10]. The inflammatory role of Panx1 channels were also seen in the present study, as treatment with Panx1-targeting nanobodies reduced serum levels of inflammatory cytokines in APAP-overdosed mice. Whereas Nb3 and Nb9 were found to elicit pronounced anti-inflammatory responses, the anti-inflammatory effects of Nb1 treatment on serum cytokine levels was less evident. The latter was more consistent with the outcome of mice treated with NAC, being the currently used antidote for clinical treatment of APAP overdosing. Indeed, NAC is widely applied as neutralizing agent and reduces hepatic injury and hepatotoxicity in patients overdosed with APAP [59]. The detoxifying effects of NAC result from its ability to replete glutathione and support mitochondrial metabolism [5]. In order to elucidate the mechanisms underlying the protective effects of the Panx1-targeting nanobodies, we measured expression levels of NLRP3 inflammasome components in the liver. In line with previous reports showing that NLRP3 is a key player in murine APAP-induced liver injury [60–63], we showed activation of the NLRP3 inflammasome pathway in APAP-treated mice. Identical to the effects observed in NAC-treated mice, treatment with Nb1 caused a reduction in the hepatic expression levels of NLRP3 and IL-1β compared to APAP-overdosed animals. On the other hand, application of Nb3 and Nb9 affected CYP2E1 and Panx1 protein expression in the liver. Histological evaluation of liver tissue also demonstrated that the Panx1-targeting nanobodies significantly reduced liver damage following APAP overdosing. Together with the reduced expression levels of serum cytokines observed in nanobody-treated animals, this suggests that Panx1-targeting nanobodies mediate their anti-inflammatory effects by affecting NLRP3 inflammasome components.

In summary, this study introduced for the first time specific, potent and in vivo-applicable nanobody-based inhibitors of Panx1 channels. As demonstrated for the case of liver disease, the Panx1-targeting nanobodies hold great promise as anti-inflammatory agents, yet this should be further tested for extrahepatic inflammatory disorders. Moreover, the Panx1-targeting nanobodies represent novel tools for fundamental research regarding the role of Panx1 channels in pathological and physiological processes.

## Conclusions

In conclusion, the de novo generated Panx1-targeting nanobodies are able to alleviate APAP-induced toxicity in mice, whereby Nb3 and Nb9 outperform NAC in terms of anti-inflammatory capacity. We showed that the protective effects of all 3 Panx1-targeting nanobodies are closely related to their ability to target the extracellular loop regions of the Panx1 protein. This corresponds with blocking potential and ultimately affects NLRP3 inflammasome components.

### Supplementary Information

**Additional file 1. Figure S1:** Immunoblot analysis of Panx1 expression following transduction of DUBCA cells. **a** DUBCA cells were transduced with lentiviral vectors to express mouse Panx1 (mPanx1) or human Panx1 (hPanx1). Protein levels of Panx1 were assessed by immunoblot analysis. Representative data of 3 independent experiments. **b** Panx1 protein levels were normalised against the total protein content and expressed as relative alteration compared to untransduced DUBCA cells. (n=3 independent experiments). All data was analysed by unpaired t-tests with Welch’s correction. Data were expressed as means ± S.D. **Figure S2:** Immunocytochemistry analysis of Panx1 expression following transduction of DUBCA cells**.** DUBCA cells were transduced with lentiviral vectors to express mouse Panx1 (mPanx1) or human Panx1 (hPanx1). DUBCA wild-type (WT), DUBCA mPanx1 and DUBCA hPanx1 cells were subjected to (1) immunocytochemistry analysis of Panx1 (red) with nuclear counterstaining (blue). Scale bar represents 500 µm (green). For (2) negative controls, the primary antibody directed against Panx1 was omitted. Representative data of 3 independent experiments. **Figure S3:** *In vivo* biodistribution of Panx1-targeting nanobodies. Nb1, Nb3, Nb9 and non-targeting Nb were radiolabeled with Technetium-99m (^99m^Tc), and 5 µg of radiolabeled nanobody was injected intravenously in healthy adult mice. Biodistribution of nanobodies was determined by γ-counting of isolated organs and expressed as percentage of injected activity per gram of organ (n=3 animals per group). Data were expressed as means ± S.D. **Figure S4:** Analysis of necrosis in acetaminophen-overdosed mice. Adult mice were overdosed with acetaminophen (APAP) (300 mg/kg) or kept untreated (UTC). After 2 hours, some mice were additionally administered either nanobody (Nb1, Nb3, Nb9 or non-targeting Nb) (10 mg/kg) or *N*-acetylcysteine (NAC) (200 mg/kg). Sampling was performed 24 hours after APAP overdosing. The percentage of necrosis was determined by measuring areas of necrosis on 10 µm liver sections stained with hematoxylin and periodic acid Schiff base (H-PAS) (n=4 (UTC and APAP) or n=12 (Nb1, Nb3, Nb9, non-targeting Nb and NAC) animals per group). All data was analysed by parametric 1-way analysis of variance followed by post hoc tests with Bonferroni’s correction. Data were expressed as means ± S.D.

## Data Availability

The data that support the findings of this study are available from Mathieu Vinken upon reasonable request.

## Abbreviations

APAP: Acetaminophen
ATP: Adenosine triphosphate
BCA: Bicinchoninic acid
CYP: Cytochrome
DILI: Drug-induced liver injury
Kd: Equilibrium dissociation constant
EDTA: Ethylenediaminetetraacetic acid
FBS: Fetal bovine serum
H-PAS: Hematoxylin and periodic acid Schiff base
HEK: Human embryonal kidney
hPanx1: Human Panx1
IMAC: Immobilized metal affinity chromatography
IL: Interleukin
IPTG: Isopropyl-β-D-thiogalactoside
MCP-1: Monocyte chemoattractant protein-1
mPanx1: Mouse Panx1
NAC: *N*-Acetylcysteine
NAPQI: *N*-Acetyl-*p*-benzoquinone imine
Panx1: Pannexin1
PEI: Polyethylenimine
RIPA: Radioimmunoprecipitation
SDS: Sodium dodecyl sulphate
99mTc: Technetium-99m
UTC: Untreated control
WT: Wild-type
