# Supplementary material for: Nanobody-based pannexin1 channel inhibitors reduce inflammation in acute liver injury
Source: J Nanobiotechnology. 2023 Oct 11;21:371. doi: 10.1186/s12951-023-02137-1 (PMC10566086; doi:10.1186/s12951-023-02137-1)
Supplement: Supplementary file 1 — Additional file 1. Figure S1: Immunoblot analysis of Panx1 expression following transduction of DUBCA cells. a DUBCA cells were transduced with lentiviral vectors to express mouse Panx1 (mPanx1) or human Panx1 (hPanx1). Protein levels of Panx1 were assessed by immunoblot analysis. Representative data of 3 independent experiments. b Panx1 protein levels were normalised against the total protein content and expressed as relative alteration compared to untransduced DUBCA cells. (n=3 independent experiments). All data was analysed by unpaired t-tests with Welch’s correction. Data were expressed as means ± S.D. Figure S2: Immunocytochemistry analysis of Panx1 expression following transduction of DUBCA cells. DUBCA cells were transduced with lentiviral vectors to express mouse Panx1 (mPanx1) or human Panx1 (hPanx1). DUBCA wild-type (WT), DUBCA mPanx1 and DUBCA hPanx1 cells were subjected to (1) immunocytochemistry analysis of Panx1 (red) with nuclear counterstaining (blue). Scale bar represents 500 µm (green). For (2) negative controls, the primary antibody directed against Panx1 was omitted. Representative data of 3 independent experiments. Figure S3: In vivo biodistribution of Panx1-targeting nanobodies. Nb1, Nb3, Nb9 and non-targeting Nb were radiolabeled with Technetium-99m (99mTc), and 5 µg of radiolabeled nanobody was injected intravenously in healthy adult mice. Biodistribution of nanobodies was determined by γ-counting of isolated organs and expressed as percentage of injected activity per gram of organ (n=3 animals per group). Data were expressed as means ± S.D. Figure S4: Analysis of necrosis in acetaminophen-overdosed mice. Adult mice were overdosed with acetaminophen (APAP) (300 mg/kg) or kept untreated (UTC). After 2 hours, some mice were additionally administered either nanobody (Nb1, Nb3, Nb9 or non-targeting Nb) (10 mg/kg) or N-acetylcysteine (NAC) (200 mg/kg). Sampling was performed 24 hours after APAP overdosing. The percentage of necrosis was determined [file 12951_2023_2137_MOESM1_ESM.docx]

**Nanobody-based pannexin1 channel inhibitors reduce inflammation in acute liver injury**

Raf Van Campenhout^1,$^, Timo W.M. De Groof^2,$^, Prashant Kadam^1^, Brenda R. Kwak^3,4^, Serge Muyldermans^5^, Nick Devoogdt^2,*^ and Mathieu Vinken ^1,*^

**Additional figures**

a

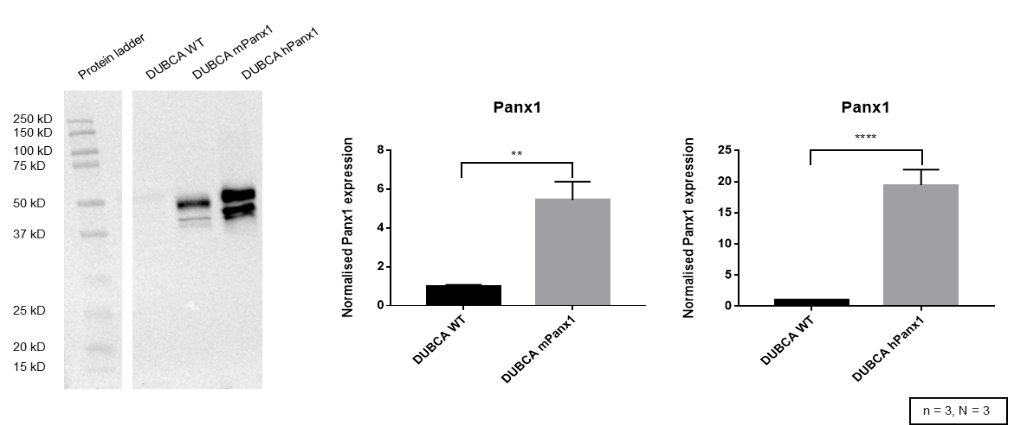


b


**Additional file 1: Figure S1: Immunoblot analysis of Panx1 expression following transduction of DUBCA cells.** **a)** DUBCA cells were transduced with lentiviral vectors to express mouse Panx1 (mPanx1) or human Panx1 (hPanx1). Protein levels of Panx1 were assessed by immunoblot analysis. Representative data of 3 independent experiments. **b)** Panx1 protein levels were normalised against the total protein content and expressed as relative alteration compared to untransduced DUBCA cells. (n=3 independent experiments). All data was analysed by unpaired t-tests with Welch’s correction. Data were expressed as means ± S.D.


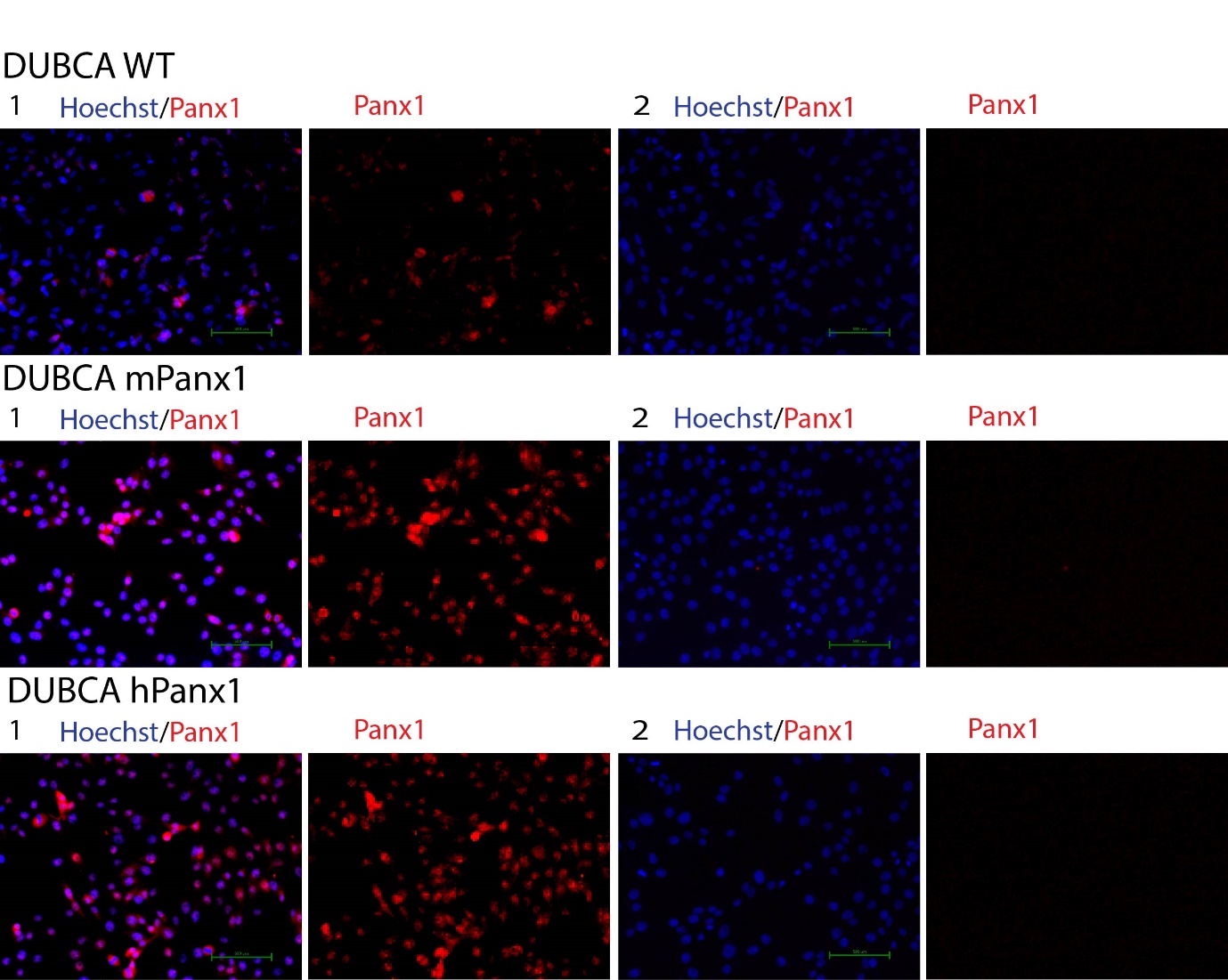


**Additional file 1: Figure S2: Immunocytochemistry analysis of Panx1 expression following transduction of DUBCA cells.** DUBCA cells were transduced with lentiviral vectors to express mouse Panx1 (mPanx1) or human Panx1 (hPanx1). DUBCA wild-type (WT), DUBCA mPanx1 and DUBCA hPanx1 cells were subjected to (1) immunocytochemistry analysis of Panx1 (red) with nuclear counterstaining (blue). Scale bar represents 500 µm (green). For (2) negative controls, the primary antibody directed against Panx1 was omitted. Representative data of 3 independent experiments.

**Additional file 1: Figure S3: *In vivo* biodistribution of Panx1-targeting nanobodies.** Nb1, Nb3, Nb9 and non-targeting Nb were radiolabeled with Technetium-99m (^99m^Tc), and 5 µg of radiolabeled nanobody was injected intravenously in healthy adult mice. Biodistribution of nanobodies was determined by γ-counting of isolated organs and expressed as percentage of injected activity per gram of organ (n=3 animals per group). Data were expressed as means ± S.D.

**Additional file 1: Figure S4: Analysis of necrosis in acetaminophen-overdosed mice.** Adult mice were overdosed with acetaminophen (APAP) (300 mg/kg) or kept untreated (UTC). After 2 hours, some mice were additionally administered either nanobody (Nb1, Nb3, Nb9 or non-targeting Nb) (10 mg/kg) or *N*-acetylcysteine (NAC) (200 mg/kg). Sampling was performed 24 hours after APAP overdosing. The percentage of necrosis was determined by measuring areas of necrosis on 10 µm liver sections stained with hematoxylin and periodic acid Schiff base (H-PAS) (n=4 (UTC and APAP) or n=12 (Nb1, Nb3, Nb9, non-targeting Nb and NAC) animals per group). All data was analysed by parametric 1-way analysis of variance followed by post hoc tests with Bonferroni’s correction. Data were expressed as means ± S.D.
